# Predicting the risk of mental disorders using complete blood count indicators: a machine learning approach

**DOI:** 10.3389/fmed.2026.1697770

**Published:** 2026-03-11

**Authors:** Yuan Su, Qi Xiong, Xintong Wei, Kaikai Jia, Chao Xie, Yuanjun Zou

**Affiliations:** School of Medical Information, Changchun University of Chinese Medicine, Changchun, China

**Keywords:** complete blood count indicators, machine learning, mental disorders, nomogram, predictive model

## Abstract

**Background:**

This study aims to explore the use of readily available complete blood count (CBC) indicators, combined with machine learning algorithms, to build a predictive model for mental disorders.

**Methods:**

This study recruited 1,379 university volunteers in September 2024, collecting data on age, gender, and 22 CBC variables. The dependent variable was a binary outcome assessed by the university’s mental health evaluation system based on the SCL-90 scale, consisting of a positive group with mental disorders and a negative group without mental disorders. SMOTETomek hybrid sampling was applied to resolve data imbalance. Random Forest (RF) was used for feature selection. This study then constructed and compared four machine learning models: eXtreme Gradient Boosting (XGBoost), Adaptive Boosting (AdaBoost), Random Forest (RF), and Gradient Boosting Decision Tree (GBDT). Model performance was evaluated using AUC, F1-score, accuracy, sensitivity, and specificity. The Shapley Additive exPlanations (SHAP) method was employed to interpret the optimal model. Furthermore, a logistic regression (LR) algorithm was used to build a nomogram.

**Results:**

Among the 1,379 volunteers, 1,023 tested negative and 356 tested positive. Fifteen volunteers had missing data for four indicators. Feature selection based on the random forest method identified 14 optimal variables for model construction. Among the six machine learning algorithms tested, XGBoost demonstrated the best performance with the highest AUC, reaching 0.860 on the training set and 0.827 on the testing set. A SHAP analysis of the XGBoost model and the nomogram results both confirmed that the top three contributing features were Basophil Percentage (BASO%), Basophil Count (BASO#), and Mean Corpuscular Hemoglobin (MCH).

**Conclusion:**

This study successfully developed a mental disorders prediction model based on the XGBoost algorithm and complete blood count data, providing clinicians with objective risk assessment indicators to assist in diagnosis and improve both efficiency and accuracy.

## 1 Introduction

Mental disorders (MD), often referred to as psychiatric disorders, are conditions that affect emotions, cognition, and regulatory functions (1). As fast-paced, high-pressure lifestyles become more common, the prevalence of mental disorders has increased dramatically. This trend has emerged as a key social issue and presents a major challenge to public health. The Global Burden of Disease Study 2021 reported that from 1990 to 2021, the number of MD cases reached 444 million, with disability-adjusted life years attributable to MD increasing by 17.28% to a total of 155 million. Consequently, the global burden ranking of MD rose from 13th in 1990 to 7th in 2019, indicating a rapid increase in its proportion of the global disease burden (2). Furthermore, the outbreak of COVID-19 further exacerbated this trend. Studies have shown a significant increase in the global incidence of mental disorders during the pandemic, with the prevalence of depression, anxiety, post-traumatic stress disorder, and insomnia rising to 28.5, 28.7, 25.5, and 24.4%, respectively (3). However, the clinical diagnosis of MD currently primarily relies on professionals using the criteria listed in the Diagnostic and Statistical Manual of Mental Disorders, Fifth Edition (DSM-5) (4), and assessments are conducted through scales or interviews, which are largely subjective. Therefore, the lack of objective biomarkers for assessing mental disorders is a critical issue, which greatly reduces the efficiency and accuracy of diagnosis. Although many researchers have made considerable efforts, no reliable biomarkers have yet been identified (5, 6). Stefan Harsanyi et al. (5) conducted a retrospective analysis of numerous studies to explore the role of cytokines and inflammation in depression, and reviewed these biological indicators as potential biomarkers. Francesca Martella et al. (6) conducted a retrospective analysis of previous studies and combined imaging techniques with genetic information to identify biomarkers for MD. However, these findings indicate that discovering stable and reliable biomarkers remains an unresolved challenge.

CBC is a routine blood test that reflects the quantity, proportion, and morphology of various blood cells, playing a crucial role in assessing an individual’s overall health (7). In recent years, with the development of artificial intelligence and machine learning technologies, many studies have been exploring how to use easily accessible blood test data, such as CBC, to predict diseases. Studies have shown that certain CBC variables are associated with various diseases. In the field of cardiovascular diseases, changes in white blood cell count are linked to an increased risk of developing cardiovascular disease (8). For metabolic diseases, changes in parameters such as neutrophil, lymphocyte, and platelet counts have been proven to be effective markers for predicting prediabetes and diabetes (9). Furthermore, the potential role of CBC indicators in MD research has also garnered significant attention. Fusar-Poli et al. (10) analyzed CBC markers from 371 patients with MD and found that the platelet-lymphocyte ratio may be associated with a specific state of bipolar disorder. These findings offer new insights into using quantifiable biological indicators to assist in MD diagnosis.

Machine learning (ML) has emerged as a crucial tool in public health research, providing effective solutions to numerous problems in this field (11, 12). Morel et al. (13) utilized a large-scale real-world dataset to develop and evaluate two machine learning algorithms for predicting psychiatric patient readmission rates. The results showed that the XGBoost algorithm performed superiorly, holding potential for reducing readmissions among psychiatric patients. Haque et al. (14) analyzed a large dataset to develop an application for detecting adolescent MD, which aims to assist parents and teachers in the early identification of these conditions. Currently, significant progress has been made in predicting MD by combining ML with multiple data sources. For instance, Saito et al. (15) analyzed wearable device and routine physical examination data from 4,612 participants to predict individuals with MD, identifying key features such as sleep quality, drinking habits, and triglycerides. Similarly, Banerjee et al. (16) analyzed foundational data on 3,532 participants, including demographic characteristics, occupational status, and lifestyle, and applied ML methods to predict the risk of MD.

In the field of disease detection, different types of machine learning algorithms possess distinct characteristics, and their applications are adaptable to diverse clinical data scenarios (17). Ensemble learning algorithms represent one of the most widely applied methodologies in contemporary clinical prediction, primarily enhancing predictive robustness through “multi-model fusion.” XGBoost and GBDT evolved from the gradient boosting framework. The XGBoost algorithm incorporates a regularization term to mitigate overfitting, while GBDT employs gradient descent for core error adjustment. Adaboost focuses on small samples through dynamic weighting adjustments, and the RF algorithm reduces variance in individual trees via bootstrap sampling and random feature selection. This class of algorithms demonstrates strong adaptability to data with multiple features and small sample sizes. However, training such models is relatively time-consuming, and hyperparameter tuning requires optimization through cross-validation. As a classic binary classification algorithm, SVM addresses high-dimensional classification problems through kernel function mapping. However, it is sensitive to noisy data and may exhibit classification bias when handling imbalanced datasets. In the medical field, it is well-suited for screening small-sample data in the early stages of disease detection. The MLP algorithm is a feedforward artificial neural network whose core function is to automatically select features through nonlinear mapping across multiple layers of neurons. It is well-suited for uncovering latent nonlinear relationships between indicators and diseases, but it is best suited for training with large datasets and is prone to overfitting.

However, the predictor variables used in the aforementioned studies (daily activities, clinical information, etc.) face limitations in accessibility. In contrast, blood count data, due to its low cost and ease of acquisition, offers the potential for constructing simpler and more practical MD prediction models. Simultaneously, drawing on the comparative analysis of the aforementioned machine learning algorithms, this study aims to construct an MD prediction model using readily available blood routine data. This model serves as an effective supplement to traditional diagnostic methods, enhancing medical efficiency and patient compliance through this low-cost approach.

## 2 Materials and methods

### 2.1 Study design

**FIGURE 1:**
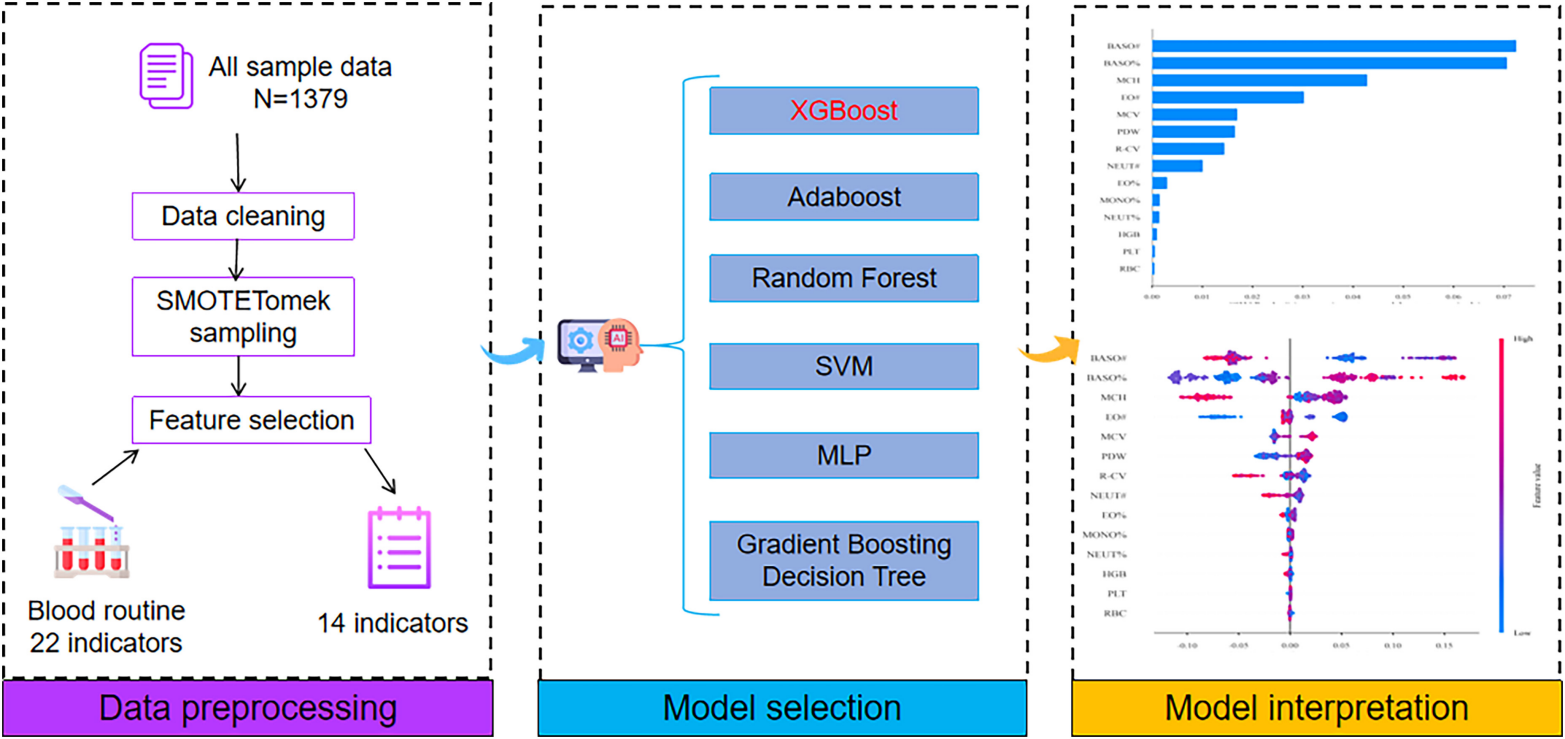
Research framework diagram.

This study was designed as a cross-sectional observational study and was conducted and reported in strict accordance with the STROBE guidelines to ensure the transparency and methodological rigor of the research. The model developed in this study leverages CBC indicators from both healthy individuals and patients with MD to predict the risk of MD. Furthermore, the SHAP algorithm was applied to quantify the influence of each CBC indicators on the prediction outcome, thereby enhancing the model’s interpretability. Ultimately, the model is designed to serve as an auxiliary tool for clinicians, helping them better identify at-risk individuals and facilitate the early management of MD. The research framework is shown in Figure 1.

### 2.2 Data acquisition

This study recruited 1,379 first-year student volunteers from a university in September 2024. The volunteers’ 22 CBC indicators were obtained from physical examination records at the Third Affiliated Clinical Hospital of Changchun University of Chinese Medicine, and their psychological test results were acquired from the university’s psychological assessment system. This study was conducted in accordance with the Declaration of Helsinki, and all participants provided written informed consent. A total of 24 variables were included in the study as independent variables, comprising gender, age, and 22 CBC indicators. The blood routine data includes Red Blood Cell Count (RBC),Mean Corpuscular Volume (MCV), Platelet Distribution Width (PDW), White Blood Cell Count (WBC), Neutrophil Percentage (NEUT%), Lymphocyte Percentage (LYMPH%), Eosinophil Percentage (EO%), Basophil Percentage (BASO%), Neutrophil Count (NEUT#), Eosinophil Count (EO#), Lymphocyte Count (LYMPH#), Basophil Count (BASO#), Hemoglobin (HGB), Hematocrit (HCT), Mean Corpuscular Hemoglobin (MCH), Mean Corpuscular Hemoglobin Concentration (MCHC), Red Cell Distribution Width-Coefficient of Variation (R-CV), Platelet Count (PLT), Mean Platelet Volume (MPV), Plateletcrit (PCT), Monocyte Count (MONO#), Monocyte Percentage (MONO%). Among the data obtained from 1,379 volunteers, 14 cases were missing for PDW, MPV, and PCT indicators, and 1 case was missing for the R-CV indicator. In this study, the dependent variable was defined as a binary variable based on the SCL-90 Symptom Checklist 90, which was generated by the university’s mental health assessment system. This variable was used to classify participants into two groups: The positive group with various mental disorders, and the negative group with no mental disorders.

### 2.3 Data preprocessing

Before model construction, the collected dataset was cleaned. This dataset comprised 356 positive samples (MD group) and 1,023 negative samples (non-MD group). Data preprocessing was performed separately on the positive and negative groups of the dataset using Python (version 3.10.2) to remove redundant information, including outliers and missing values, thereby ensuring data quality and accuracy. For outlier handling, the Interquartile Range (IQR) method was used to identify outliers, and these values were replaced with the mode of the respective variable to mitigate the impact of extreme values on model stability. For missing data, the k-nearest neighbors (KNN) imputation method was employed to fill the missing values, thereby reducing the bias caused by incomplete information. Subsequently, the dataset was randomly split into a training set (80%) and a testing set (20%), with a random seed set to 42 to ensure reproducibility of the random process. The training set was used for model development, while the testing set was used to evaluate the model’s performance and generalizability. Continuous variables were standardized, and categorical variables such as gender were one-hot encoded to minimize the impact of differences in variable units and scales on the analysis. In this study, positive samples for MD accounted for 25.82% (356/1,379), while negative samples comprised 74.18% (1,023/1,379). Since the ratio of positive to negative samples is approximately 1:3, there is a clear data imbalance problem, which may lead to overfitting during model training. To address this issue, we employed the SMOTETomek hybrid sampling method. The SMOTE algorithm performs oversampling on the minority class by generating new synthetic samples through interpolation between neighboring minority-class instances, thereby alleviating class imbalance (18). The Tomek Links undersampling algorithm performs undersampling on the majority class by removing majority-class samples that form “Tomek links,” which reduces interference from the majority class on the minority class (19). The combination of SMOTE oversampling for the minority class and Tomek Links undersampling for the majority class both increases the number of minority-class samples and cleans the sample space of the majority class. This combined approach mitigates class imbalance more effectively than single sampling methods and reduces overfitting. It is widely used to address data imbalance and ambiguous class boundaries. This technique combines the minority class oversampling of SMOTE with the majority class under sampling of Tomek Links, and is commonly used to resolve issues of data imbalance and fuzzy class boundaries. In the medical field, this hybrid sampling method is also widely used, helping models more accurately distinguish between healthy and patient populations (20, 21). This study selected the RF algorithm for feature selection because it can capture nonlinear and complex feature interactions and effectively identify the variables that contribute most to prediction in high-dimensional and noisy data (22).

### 2.4 Machine learning algorithms

#### 2.4.1 XGBoost algorithm

It is an efficient and flexible gradient boosting decision algorithm, as shown in Equations 1, 2. It sequentially trains models to correct the errors of its predecessors and incorporates regularization techniques during the training and ensemble process, making it an ideal choice for both classification and regression tasks (23, 24). The mathematical expression is


$$y_{i}^{\wedge }\,\left(t\right)=\sum_{m=1}^{t}f_{m}\,\left(x_{i}\right)=y_{i}^{\wedge }\,\left(t-1\right)+f_{t}\,\left(x_{i}\right)$$ (1)

In the equation, m denotes the mth tree, x_i_ represents all features of the ith sample, $y_{i}^{\wedge }\,\left(t\right)$is the predicted value of the ith sample after t iterations, and f_m_(x_i_) is the predicted value of the ith sample by the mth tree. The mathematical expression is


$$\mathrm{obj}=\sum_{i=1}^{n}l\,\left(y_{i},y_{i}^{\wedge }\right)+\sum_{m=1}^{t}\Omega \,\left(f_{m}\right)$$ (2)

In the formula, $l\,\left(y_{i},y_{i}^{\wedge }\right)$ describes the discrepancy between the predicted value and the actual value, while Ω(*f*_*m*_) prevents overfitting.

#### 2.4.2 GBDT algorithm

It is an iterative approach that sequentially builds decision trees, with each tree correcting the errors of the previous one, as shown in Equations 3–7. It’s a versatile algorithm that’s commonly used for both classification and regression tasks (25, 26). The specific steps of the algorithm are as follows:

(1) Initialization function to minimize the loss function:
$$f_{0}\,\left(x\right)=a\,r\,g\,m\,i\,n\,\sum_{i=1}^{N}L\,\left(y_{i},c\right)$$ (3)

In the equation, f_0_(x) is a tree with only one root node, L(y_i_,c) is the loss function, and c is the constant that minimizes the loss function.

(2) Iterative training. Let the iteration count be m = 1, 2, …, M.

① For samples i = 1,2,…,N, compute the negative gradient of the loss function as the residual estimate:


$$r_{m\,i}=-\left[\frac{\partial L\,\left(y_{i},f\,\left(x_{i}\right)\right)}{\partial f\,\left(x_{i}\right)}\right]\,f\,\left(x\right)=f_{m-1}\,\left(x\right)$$ (4)

② Fit a regression tree using r_*mi*_ to obtain the leaf node regions (j = 1,2,…,J) for the mth tree.

③ Estimate the area value of leaf nodes using linear search to minimize the loss function:


$$c_{m\,j}=a\,r\,g\,m\,i\,n\,\sum_{x_{i}\in R_{m\,j}}{\left(y_{i}-\left(f_{m-1}\,\left(x_{i}\right)+c\right)\right)}^{2}$$ (5)

④ Update the learner, where I denotes the indicator function:


$$f_{m}\left(x\right)=f_{m-1}\left(x\right)+\sum_{m=1}^{M}c_{m\,j}\cdot I\left(x\in R_{m\,j}\right)$$ (6)

(3) Sum all iteration nodes to obtain the final regression model:
$$f_{m}^{\wedge }\left(x\right)=f_{M}\left(x\right)=\sum_{m=1}^{M}\sum_{j=1}^{J}c_{m\,j}\cdot I\left(x\in R_{m\,j}\right)$$ (7)

#### 2.4.3 AdaBoost algorithm

It is a boosting-based ensemble learning method that works by iteratively adjusting the weights of misclassified samples, as shown in Equations 8–12. It then linearly combines these weak learners to form a single strong learner (27). The algorithm steps are as follows:

(1) () Initialize sample weights. Select n groups of sample data from the dataset and initialize the sample weights.
W⁢(i)=1/n (8)
(2) Iteratively train the weak learner model. *e_t_* denotes the sum of prediction errors for sequence *r_t_*.
$$e_{t}=\sum_{i}W_{t}\,\left(i\right),i=1,2,\ldots \,n$$ (9)
(3) Calculate model weights. Calculate the weights of *b_t_* based on *e_t_*.
$$b_{t}=\frac{1}{2}\,l\,n\,\left(\frac{1-e_{t}}{e_{t}}\right)$$ (10)
(4) Update the weights of the samples.
$$W_{t+1}\,\left(i\right)=\frac{W_{t}\,\left(i\right)}{B_{i}}\cdot e^{\left[-b_{t}\cdot y_{i}\cdot r_{t}\,\left(x_{i}\right)\right]}$$ (11)
(5) Deep learning model. After training N weak learners, we obtain N sets of prediction functions f(r_t_,b_t_). Combining these yields the strong learner prediction model h(x).
$$h\,\left(x\right)=\sum_{i=1}^{N}\left(\ln\,\frac{1}{a_{t}}\right)*f\,\left(r_{t},b_{t}\right)$$ (12)

#### 2.4.4 RF algorithm

It is an ensemble method that combines multiple decision trees to optimize feature utilization and reduce the impact of noisy features (22), as shown in Equation 13. The algorithm generates training subsets through sampling with replacement. Each subset trains a complete decision tree. When new data is input, all trees make independent predictions. The average of these predictions serves as the final regression value. The regression prediction expression for RF is as follows:


$$y^{\wedge }=\frac{1}{T}\,\sum_{j=1}^{T}\left\{F\,\left(x,\theta_{t}\right)\right\}$$ (13)

In the formula, *y*^∧^ represents the model’s predicted value, T denotes the number of decision trees, F(x,θ_t_) indicates multiple decision trees, x refers to the individual input features, and θ_t_ denotes the random variables in each tree that follow independent tests.

#### 2.4.5 SVM algorithm

SVM is a binary classification model. This algorithm finds the optimal hyperplane to separate data points into distinct categories, with the hyperplane determined by the support vectors (28), as shown in Equations 14–17. The hyperplane is represented as:


f⁢(x)=(w⋅x+b) (14)

In the formula, w is the adjusted weight vector, and b is the bias term. The SVM aims to maximize the margin between the two classes. Therefore, it seeks the optimal hyperplane, expressed as:


$$\min\,\frac{1}{2}\,{||w||}^{2}+c\,\sum_{i=1}^{n}\xi_{i}$$ (15)

By applying the Lagrange multiplier method, the optimal hyperplane is transformed into a quadratic programming problem with a unique solution. The formula is as follows:


$$L=\left(w,b,\xi ,a\right)=\sum_{i,j=1}^{n}a_{i}-\frac{1}{2}\,\sum_{i=1}^{n}\sum_{j=1}^{n}$$ (16)

$$a_{i}\,a_{j}\,y_{i}\,y_{j}\,\varphi \,\left(x_{i}\right)\,\varphi \,\left(y_{i}\right)$$

The classification decision function is:


$$f\,\left(x\right)=\mathrm{sign}\,\left(\sum_{i,j=1}^{n}a_{i}\,y_{i}\,\left(\varphi \,\left(x_{i}\right)\,\varphi \,\left(y_{i}\right)\right)+b\right)$$ (17)

#### 2.4.6 MLP algorithm

**FIGURE 2:**
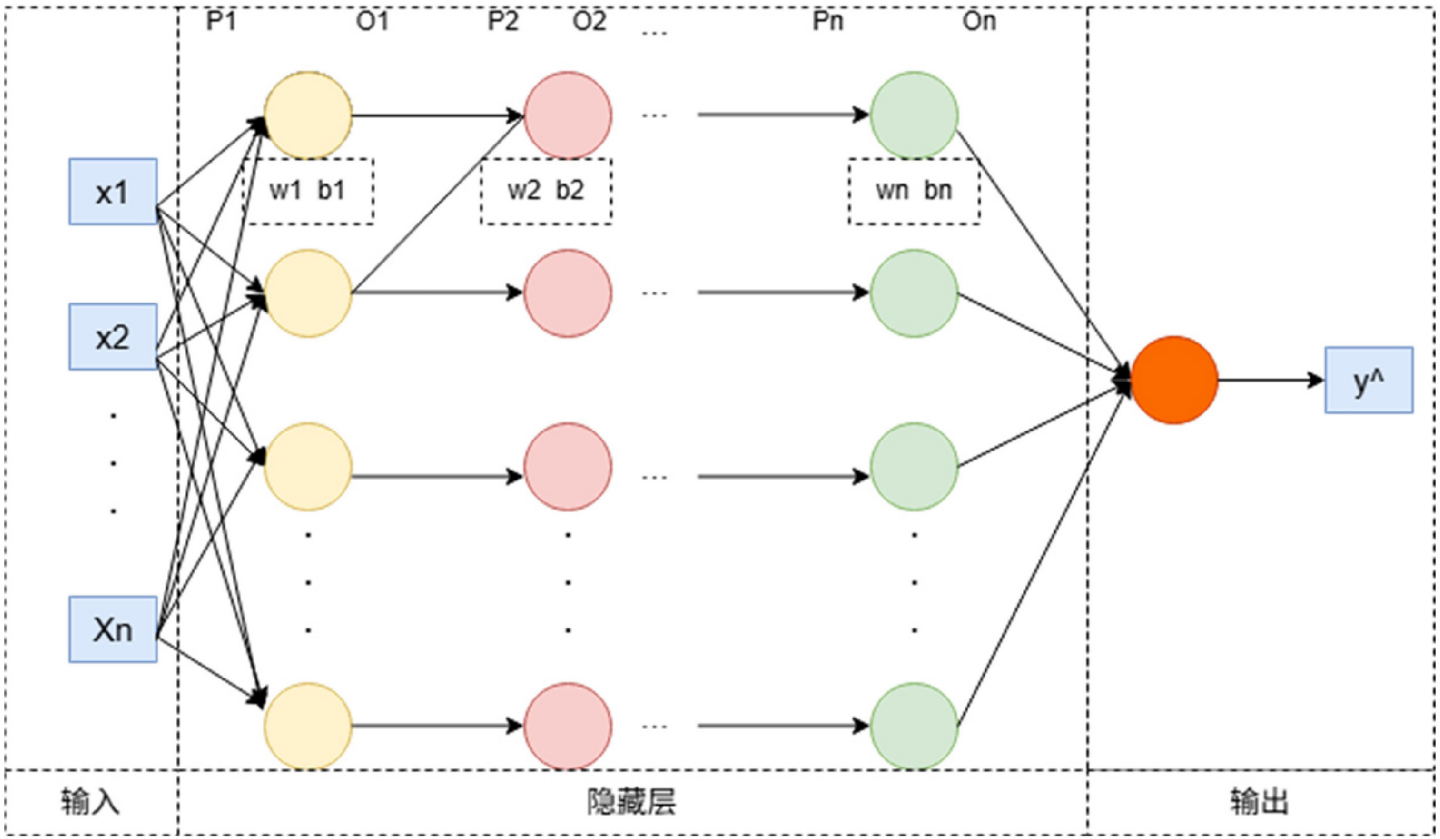
MLP model framework diagram.

MLP is a feedforward artificial neural network. It consists of multiple layers of neurons, each layer containing several neurons with nonlinear activation functions (29), as shown in Equations 18–22. The basic structure is shown in Figure 2. There are N layers in the network. w_n_, b_n_ represent the weight matrices and bias vectors of the nth and (n+1) th layers, respectively. p_n_ denotes the input to the nth layer, and o_n_ denotes the output of the nth layer. The formula for the MLP algorithm process is as follows:

The forward propagation formula is:


$$P_{n}=O_{n-1}\cdot w_{n}+b_{n}$$ (18)

$$O_{N}=f\,\left(P_{N}\right)$$ (19)

The loss function is:


$$J={\left(y-y^{\wedge }\right)}^{2}$$ (20)

The backpropagation formula is: d(w_n_) is the gradient of the loss function with respect to w_n_, d(b_n_) is the gradient of the loss function with respect to b_n_, h is the learning rate. d(o_n_) is the derivative of the activation function at layer n.


$$d\,\left(b_{n}\right)=d\,\left(b_{n+1}\right)\cdot w_{n+1}\cdot d\,\left(o_{n}\right)$$ (21)

$$d\,\left(w_{n}\right)=d\,\left(b_{n}\right)\cdot o_{n-1}$$ (22)

### 2.5 Model evaluation

In this study, five-fold cross-validation was employed to evaluate the classifier’s performance. The dataset was randomly partitioned into five equal subsets. In each iteration, one subset served as the testing set, while the remaining four subsets were used as the training set. This process was repeated five times, ensuring that each subset was used as the test set exactly once. To evaluate the model’s performance, this study used the following metrics: sensitivity (Sn), specificity (Sp), accuracy (Acc), F1-score, and AUC value (30). The formula is as follows in Equations 23–26:


Sn=TP/(TP+FN) (23)

Sp=TN/(TN+FP) (24)

Acc=(TP+TN)/(TP+FN+TN+FP) (25)

F1=2⁢T⁢P/(2⁢T⁢P+FN+FP) (26)

TP represents the number of positive samples correctly predicted as positive, TN is the number of negative samples correctly predicted as negative, FP is the number of negative samples incorrectly predicted as positive, and FN is the number of positive samples incorrectly predicted as negative. Additionally, this study utilized the Receiver Operating Characteristic (ROC) curve for a comprehensive evaluation of the model’s performance.

### 2.6 Model explanation

The SHAP algorithm is a game theory-based model interpretation method widely used in machine learning. Its core principle is to quantify the contribution of each feature to a model’s prediction by calculating its SHAP value (31, 32). Therefore, to interpret the black-box nature of our ML model, we employed the SHAP algorithm, which was implemented using Python’s shap package (version 0.48.0).

### 2.7 Statistical analysis

All analyses in this study were performed using Python (version 3.10.2) and R (version 4.4.2). For continuous variables, normally distributed data were expressed as the mean ± standard deviation, while non-normally distributed data were presented as the median (interquartile range, IQR). Categorical variables were expressed as counts and percentages. Basic characteristics and CBC indicators between the MD and non-MD groups were compared. A *t*-test or Mann-Whitney U test was used for continuous variables, and a chi-square test was used for categorical variables. *P* < 0.05 was considered statistically significant. Finally, a nomogram was constructed based on the logistic regression (LR) model using the rms package and nomogram function in R (33).

## 3 Results

### 3.1 Baseline characteristics of participants

A total of 1,379 first-year university students were included in this study. Both MD and non-MD groups were of the same age, ranging from 18 to 19 years, and had an approximate male-to-female ratio of 1:1.5. Among the 22 CBC indicators, the MD group showed significant differences from the non-MD group in four variables. Specifically, the MD group had significantly higher levels of PDW, BASO#, and MPV (*P* < 0.05), while the MCH variable was significantly lower (*P* < 0.05). The remaining 18 variables did not show any statistically significant differences. Detailed baseline characteristics are presented in Table 1.

**TABLE 1:** Baseline characteristics of participants.

Variables	MD group (*N* = 356)	Non-MD group (*N* = 1,023)	*P*
Sex [n(%)] male	147 (41.29)	381 (37.24)	0.197
Female	209 (58.71)	642 (62.76)	
Age (岁)	18.00 (18.00, 19.00)	18.00 (18.00, 19.00)	0.148
RBC [M(P_25_,P_75_), × 10^12^/L]	4.50 (4.21, 4.95)	4.51 (4.23, 4.89)	0.806
MCV [M(P_25_,P_75_),fl]	88.55 (86.10, 90.30)	87.90 (86.00, 90.20)	0.375
PDW [M(P_25_,P_75_),fl]	11.20 (10.20, 12.40)	10.80 (10.10, 11.90)	0.006
WBC [M(P_25_,P_75_), × 10^9^/L]	6.46 (5.52, 7.46)	6.58 (5.65, 7.74)	0.103
NEUT% [M(P_25_,P_75_),%]	53.80 (49.10, 58.52)	53.60 (48.90, 58.75)	0.775
LYMPH%(%)	35.93 ± 7.02	35.80 ± 7.13	0.766
EO% [M(P_25_,P_75_),%]	1.70 (1.20, 2.50)	1.60 (1.20, 2.50)	0.389
BASO% [M(P_25_,P_75_),%]	0.50 (0.38, 0.60)	0.40 (0.40, 0.60)	< 0.001
NEUT# [M(P_25_,P_75_), × 10^9^/L]	3.39 (2.79, 4.05)	3.46 (2.85, 4.27)	0.091
LYMPH# [M(P_25_,P_75_), × 10^9^/L]	2.27 (1.87,2.69)	2.29 (1.98,2.73)	0.096
BASO# [M(P_25_,P_75_), × 10^9^/L]	0.03 (0.02,0.04)	0.03 (0.02,0.04)	0.703
HGB [M(P_25_,P_75_),g/L]	134.00 (123.00, 147.00)	132.00 (123.00, 146.00)	0.746
HCT [M(P_25_,P_75_),%]	39.50 (36.50, 43.00)	39.40 (37.10, 43.00)	0.441
MCH [M(P_25_,P_75_),pg]	29.70 (29.20, 30.42)	30.00 (29.10, 30.70)	0.035
MCHC [M(P_25_,P_75_),g/L]	337.00 (332.00, 343.00)	337.00 (332.00, 343.00)	0.981
R-CV [M(P_25_,P_75_),%]	12.40 (11.90, 12.80)	12.40 (12.00, 13.00)	0.245
PLT [M(P_25_,P_75_), × 10^9^/L]	258.00 (226.00, 295.25)	261.00 (225.00, 293.50)	0.524
MPV [M(P_25_,P_75_),fl]	10.10 (9.60, 10.80)	10.00 (9.50, 10.60)	0.007
PCT [M(P_25_,P_75_),%]	0.26 (0.23, 0.30)	0.26 (0.23, 0.29)	0.598
MONO# [M(P_25_,P_75_), × 10^9^/L]	0.48 (0.41, 0.58)	0.48 (0.40, 0.59)	0.537
MONO% [M(P_25_,P_75_),%]	7.40 (6.57, 8.40)	7.40 (6.50, 8.30)	0.363
EO# [M(P_25_,P_75_), × 10^9^/L]	0.10 (0.08, 0.16)	0.11 (0.07, 0.17)	0.769

The continuous variables were expressed as mean ± standard deviation (SD) or the medians with interquartile ranges (IQRs).

**FIGURE 3:**
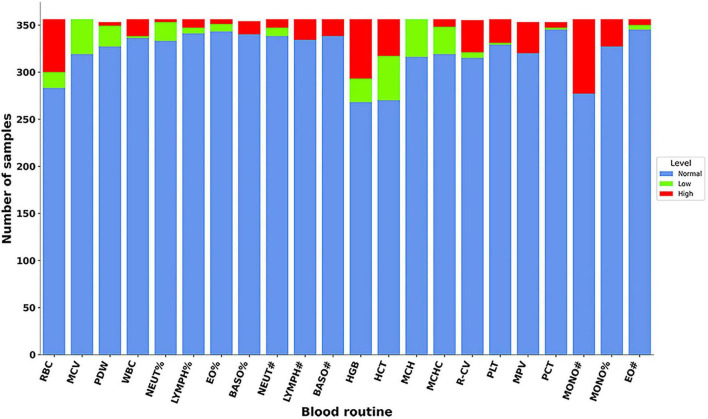
Distribution of CBC indicators in patients with MD. Comparison of patient indicators to normal reference ranges.

When the CBC indicators of MD patients were compared to the reference range, the majority of values were found to be within the normal limits. However, some individual variables in certain patients were below the reference range, including MCV, HCT, MCH, and MCHC. Additionally, some patients also presented with low RBC, HGB, and MONO# values. The detailed distribution of these variables is shown in Figure 3.

Based on the analysis, there are distinguishing variables between MD and non-MD groups. These findings provide a data foundation for the subsequent development of a ML model.

### 3.2 Feature selection

**FIGURE 4:**
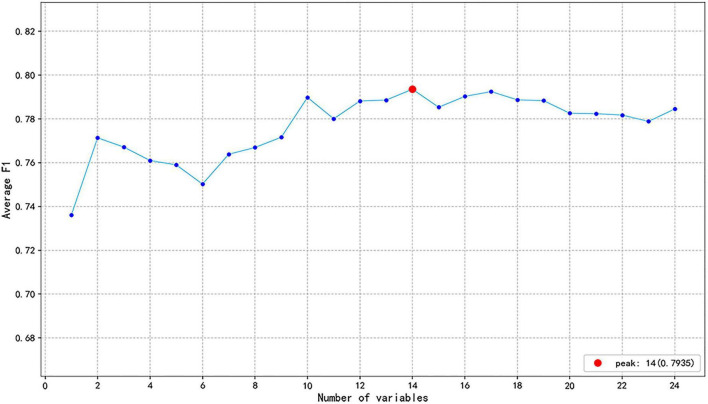
Curve of feature selection using the RF method. The F1 got the highest value when the number of variables was 14 (represented as a red solid point).

**FIGURE 5:**
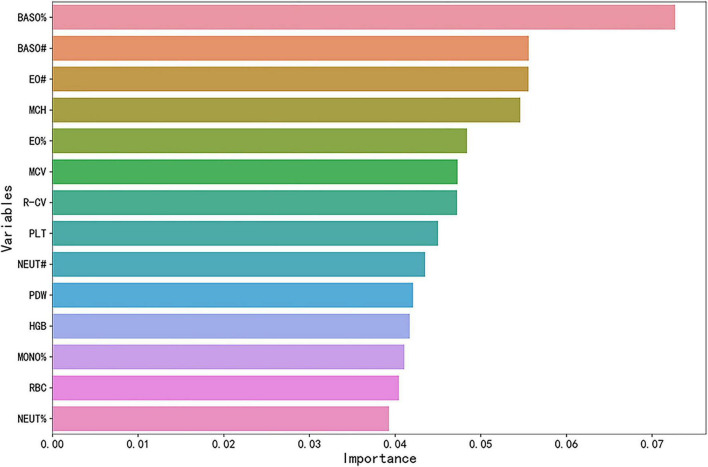
Ranking of predictive feature importance for MD patients based on the RF algorithm. The longer the bar chart, the greater the impact of the variable on the prediction results, and the more valuable it is for decision-making.

To identify key features for predicting MD, this study employed the RF algorithm to perform feature selection and calculate the contribution of the 24 variables included in the study. A ranking of these variables, based on their relevance, was obtained. As shown in Figure 4, the feature selection curve reached its peak performance when the number of variables was 14. Consequently, these 14 optimal features were selected, including RBC, MCV, PDW, NEUT%, EO%, BASO%, NEUT#, BASO#, HGB, MCH, R-CV, PLT, MONO%, and EO#. The ranking of feature importance is presented in Figure 5, with the top five variables being BASO%, BASO#, EO#, MCH, and EO%, respectively.

### 3.3 Hyperparameters for the randomizedsearchcv method

Hyperparameter selection for the six machine learning algorithms was optimized using the RandomizedSearchCV function in scikit-learn combined with five-fold cross-validation. (1) XGBoost: n_estimators = 1,000, learning_rate = 0.005, max_depth = 5, min_child_weight = 18, reg_lambda = 14, reg_alpha = 6; (2) GBDT: n_estimators = 250, max_depth = 2, learning_rate = 0.005; (3) Adaboost: n_estimators = 140, learning_rate = 0.2, estimator_max_depth = 2;(4)n_estimators = 250, max_depth = 4, max_features = 0.3; (5) SVM:C = 0.9, gamma = 0.05, kernel = “rbf”; (6) MLP: hidden_layer_sizes: (41,23), activation = “relu,” solver = “sgd,” alpha = 22.19, learning_rate = 0.001. During the random search process, these hyperparameter combinations were identified as the optimal performance combinations. Table 2 summarizes the hyperparameter selections and results for all models.

**TABLE 2:** Six machine learning algorithms for optimal random search results.

Classifier‘	Hyperparameters	Possible values	RandomizedSearchCV outcome
XGBoost	n_estimators learning_rate max_depth min_child_weight reg_lambda reg_alpha	Randint (500, 1,800) 0.005, 0.01, 0.05, 0.1 3, 4, 5, 6 10, 13, 15, 18, 20 10, 12, 14, 16, 18 2, 4, 6, 8	1,000 0.005 5 18 14 6
GBDT	n_estimators max_depth learning_rate	200, 250, 300, 350 2, 3, 4 0.001, 0.005, 0.1, 0.2	250 2 0.005
Adaboost	n_estimators learning_rate estimator_max_depth	np.arrange (50,200, 15) 0.01, 0.1, 0.2, 0.3 1, 2	140 0.2 2
RF	n_estimators max_depth max_features	200, 250, 300, 350 2, 3, 4 0.1, 0.2, 0.3, 0.4, 0.5	250 4 0.3
SVM	C gamma kernel	0.5, 0.6, 0.7, 0.8, 0.9, 1 0.01, 0.05, 0.1 rbf, sigmoid	0.9 0.05 rbf
MLP	Hidden_layer_sizes activation solver alpha learning_rate	[randint.rvs (20,100), randint.rvs (10, 25)] relu, tanh, logistic adam, sgd Uniform (2, 50) 0.0001, 0.001, 0.01, 0.1	(41,23) relu sgd 22.19 0.001

### 3.4 Construction of the MD prediction model

**FIGURE 6:**
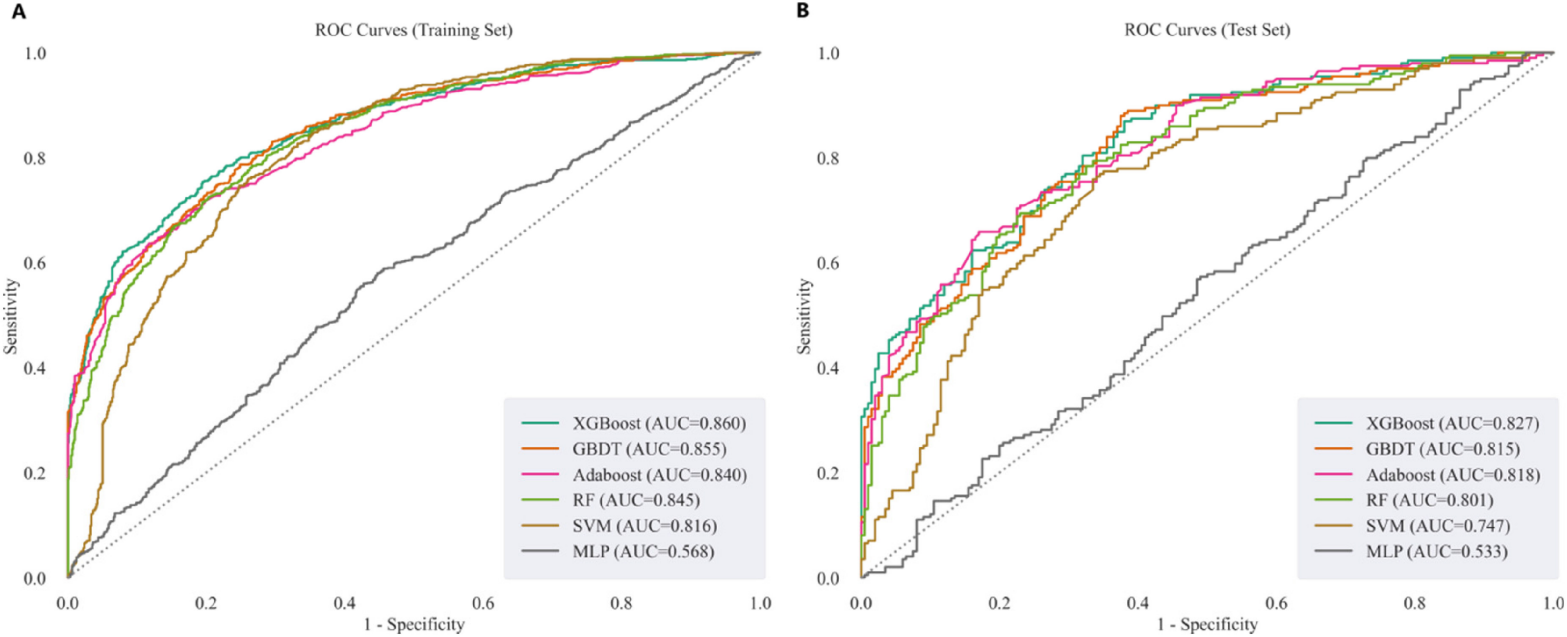
The ROC curves of different models on the training and testing sets. **(A)** ROC curves of XGBoost, GBDT, AdaBoost, RF, SVM, and MLP on the training set. **(B)** ROC curves of XGBoost, GBDT, AdaBoost, RF, SVM, and MLP on the testing set.

This study leveraged the 14 CBC indicators obtained from feature selection to construct a MD prediction model. Using this 14-dimensional dataset, six ML methods were employed to predict the probability of MD in both the training and testing sets. The optimal model parameters were determined through hyperparameter selection using the RandomizedSearchCV method and five-fold cross-validation. We evaluated model performance using AUC, F1-score, accuracy, sensitivity, specificity, and the ROC curve, ensuring a fair assessment. All four models achieved good results, with the XGBoost model outperforming the others. In the training set, XGBoost had an AUC of 0.860, while the AUC values for GBDT, AdaBoost, RF, SVM and MLP were 0.855, 0.840, 0.845, 0.816, 0.568, respectively. In the testing set, XGBoost’s AUC was 0.827, while GBDT, AdaBoost, RF, SVM and MLP had AUC values of 0.815, 0.818, 0.801, 0.747, and 0.533, respectively. Furthermore, XGBoost ranked higher than the other models in both F1-score and accuracy on both the training and testing sets. Detailed performance metrics for the training and testing sets are provided in Tables 3, 4, with the corresponding ROC curves shown in Figure 6.

**TABLE 3:** Performance evaluation of ML models on the training set.

Model	AUC	F1	Accuracy	Sensitivity	Specificity
XGBoost	0.860	0.772	0.788	0.758	0.795
GBDT	0.855	0.760	0.762	0.758	0.765
Adaboost	0.840	0.746	0.746	0.744	0.749
RF	0.845	0.759	0.756	0.770	0.741
SVM	0.816	0.770	0.705	0.847	0.646
MLP	0.568	0.573	0.556	0.590	0.529

**TABLE 4:** Performance evaluation of ML models on the testing set.

Model	AUC	F1	Accuracy	Sensitivity	Specificity
XGBoost	0.827	0.730	0.732	0.729	0.735
GBDT	0.815	0.727	0.729	0.724	0.736
Adaboost	0.818	0.726	0.719	0.744	0.700
RF	0.801	0.726	0.719	0.744	0.700
SVM	0.747	0.721	0.657	0.800	0.585
MLP	0.533	0.529	0.525	0.533	0.520

### 3.5 Evaluation of influencing factors of MD based on the SHAP method

**FIGURE 7:**
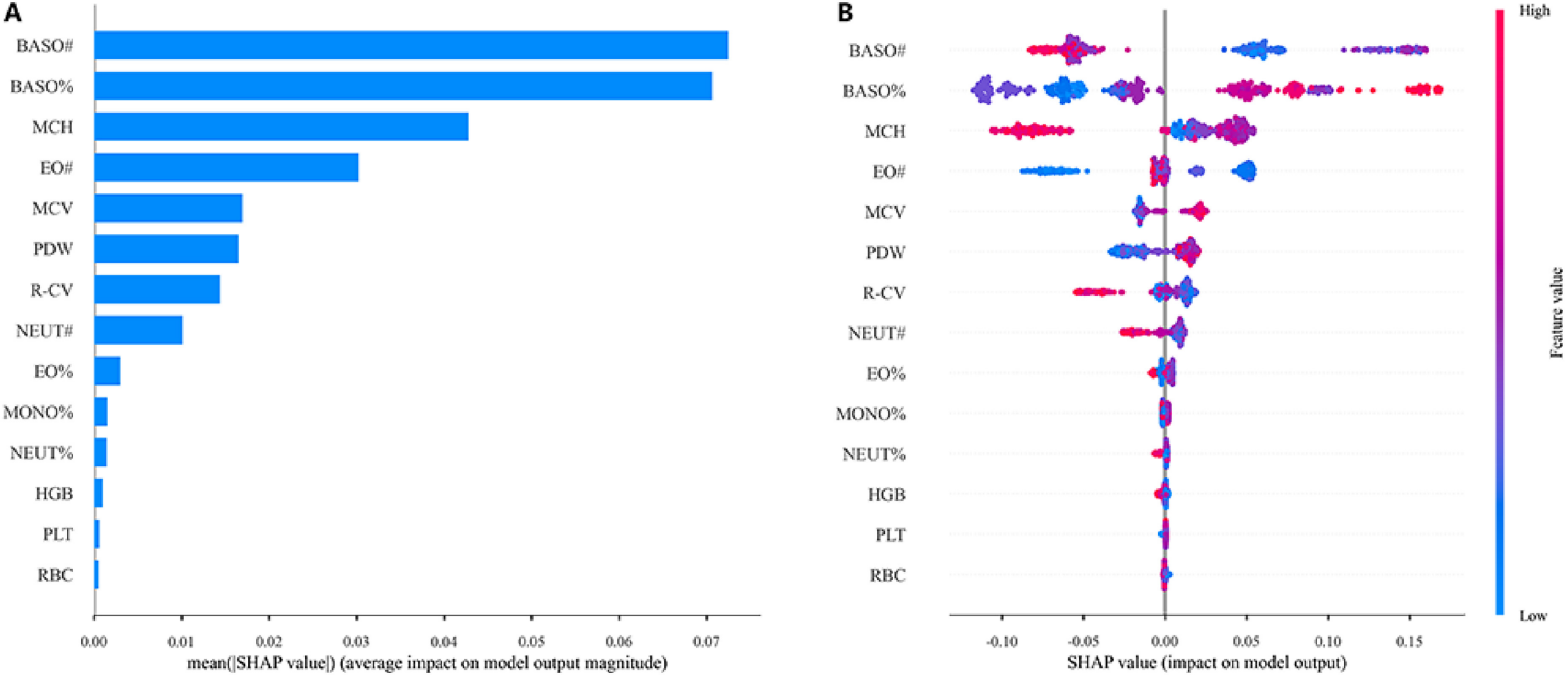
Interpretability of the XGBoost model. **(A)** Mean absolute SHAP bar plot. **(B)** The SHAP beeswarm plot.

To better understand the contribution of the 14 features to the MD prediction model, we applied the SHAP algorithm to quantify the impact of each feature. For the XGBoost model, the feature contributions are shown in Figure 6. In the mean absolute SHAP bar plot (Figure 7A), the *y*-axis lists features such as BASO#, BASO%, and MCH. The length of each bar represents the average absolute SHAP value, which indicates the average magnitude of a feature’s influence on the model’s output. The longer the bar, the greater its overall impact on the prediction. The beeswarm plot (Figure 7B) provides a more detailed view. The *y*-axis lists the 14 features, and each point in the plot represents the SHAP value for a feature in a single sample. The *x*-axis represents the SHAP value, with the following interpretations: SHAP value > 0: A specific value of the feature increases the model’s predicted output. SHAP value < 0: A specific value of the feature decreases the model’s predicted output. SHAP value ≈ 0: The feature has a negligible impact on the prediction. The color of each point indicates the original feature value, where red points signify high feature values and blue points signify low feature values.

### 3.6 Construction of the nomogram

**FIGURE 8:**
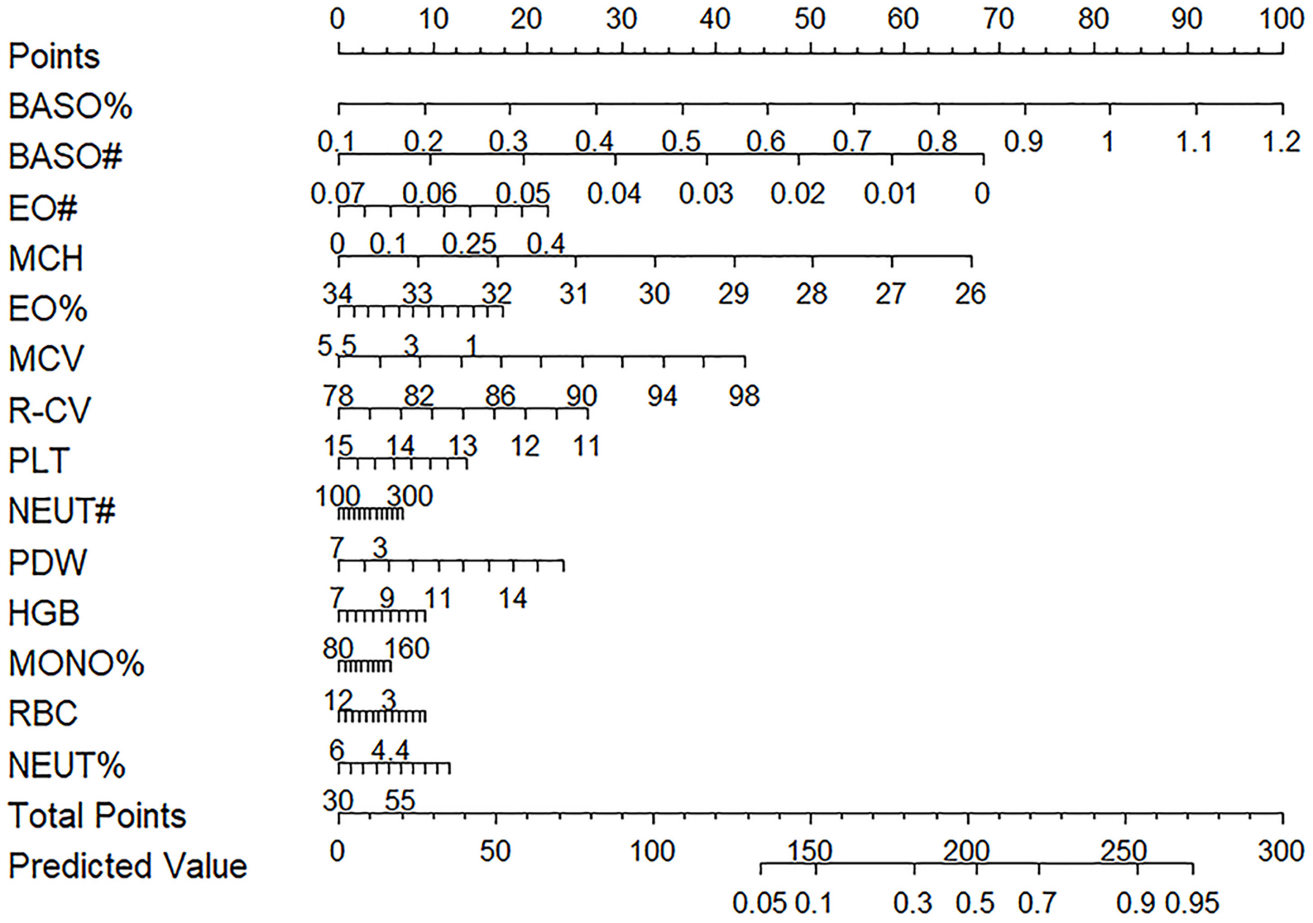
Nomogram based on the logistic regression method. The nomogram was drawn by constructing a model using logistic regression algorithm with CBC indicators as input features.

This study constructed a nomogram based on a LR model to evaluate the impact of the 14 selected variables on the prediction outcome. The visual representation of the nomogram is shown in Figure 8. In the nomogram, the length of a feature’s corresponding line segment represents its contribution to the predictive model, with a longer segment indicating a greater contribution. In this model, BASO% had the greatest contribution. The other variables, ranked from highest to lowest contribution, were BASO#, MCH, MCV, R-CV, PDW, EO#, EO%, PLT, NEUT%, HGB, RBC, NEUT#, and MONO%. The top three contributing features in this model are consistent with the results from the SHAP analysis of the XGBoost model.

## 4 Discussion

With the accelerating pace and increasing pressures of modern society, mental health issues have become increasingly prevalent, and the number of individuals with MD is showing an upward trend (3). Currently, MD is primarily diagnosed using standardized scales or clinical interviews, methods which are highly subjective and can lead to missed diagnoses. Consequently, the development of a predictive model based on objective, easily accessible, and low-cost CBC indicators holds urgent clinical value and promising application prospects for assisting physicians in MD diagnosis.

In this study, the RF algorithm was used for feature selection, identifying 14 variables for model construction. These 14 variables can be categorized into three major groups: red blood cell system, white blood cell system, and platelet system parameters. The red blood cell system parameters primarily reflect the body’s hematopoietic function. HGB and RBC are important indicators for assessing anemia (34). Anemia can lead to insufficient oxygen supply to the brain, which affects neurotransmitter synthesis and consequently increases the risk of developing MD (35). Similarly, elevated values of MCV and MCH, which are also indicators for anemia, have been associated with this condition. Research indicates that megaloblastic anemia caused by a vitamin B12 deficiency leads to an increase in MCV, thereby raising the risk of MD (36). RDW-CV reflects the red cell distribution width, and its elevation is considered a non-specific biomarker of inflammation and oxidative stress. High levels of these can damage brain cells and the blood-brain barrier, which in turn increases the risk of MD (37). The white blood cell system parameters reflect the body’s immune function and inflammatory state. Research has shown that neutrophil dysfunction in individuals with MD may lead to chronic inflammation, which can affect neurons and exacerbate the pathophysiological process of MD (38). An important factor of eosinophils, Eotaxin-1/CCL11, has been proven to damage neurons by inhibiting neurogenesis and promoting microglial activation, thereby increasing the prevalence of MD (39). Furthermore, monocytes exacerbate neuroinflammation by exhibiting dysregulation of the NF-kB and glucocorticoid response pathways, releasing pro-inflammatory cytokines and chemokines, and altering the expression of proteins (HLR-DR, TKR3 and TLR4), all of which influence the pathophysiological process of MD (40). Platelets promote the onset of MD through complex interactions with neurons, as they share key proteins such as 5-HT, BDNF, and Reelin (41). After identifying these biologically significant parameters, this study further explored how to leverage ML techniques to integrate them and establish an efficient and accurate MD diagnostic model.

In this study, we established six ML models based on CBC data to diagnose whether patients have MD. The model comparison results show that the XGBoost model demonstrated superior performance over the other three models. With AUC values of 0.858 on the training set and 0.826 on the testing set, both exceeding 80%, the XGBoost model exhibited high discriminative power and good generalization capability in distinguishing between MD and non-MD patients. XGBoost, which combines multiple decision trees, can effectively handle high-dimensional feature data. Other studies have also confirmed its significant advantages in disease prediction within the MD field. For example, Jiani Fu et al. (42) evaluated data on major depressive disorder from 124 participants using 11 predictors and demonstrated that the XGBoost model was superior to the LR, SVM, KNN, and RF algorithms. Their model achieved an AUC of 0.835, with accuracy, sensitivity, and specificity values of 0.730, 0.670, and 0.774, respectively. These findings further corroborate the value of XGBoost in MD diagnosis.

To gain further insight into the diagnostic decisions made by the XGBoost model, we conducted a detailed feature contribution analysis using the SHAP algorithm. The results showed that BASO%, BASO#, and MCH were the top three contributors to the model’s performance. This finding not only explains the key features in the model’s decision-making process but also aligns well with the known clinical and pathological characteristics of the disease, highlighting its significant clinical value. Basophils are a rare type of white blood cell primarily responsible for regulating immune and inflammatory responses and releasing mediators like histamine (43). One study found that basophils are important immune cells involved in allergic inflammatory responses, and an increase in their number can affect microglial cells and other immune cells in the central nervous system. This can worsen changes in brain function, thereby increasing the risk of MD (44). Mean Corpuscular Hemoglobin (MCH) is a crucial indicator for evaluating red blood cell morphology and protein synthesis. Research has shown that low hemoglobin levels can lead to an iron-deficient state or decreased oxygen transport capacity. This, in turn, can affect central nervous function and neurotransmitter synthesis, increasing the risk of MD (45).

To further validate our conclusions, this study constructed a nomogram based on the LR model. The nomogram analysis was highly consistent with the SHAP algorithm results, with both methods indicating that BASO%, BASO#, and MCH are the key factors influencing the diagnostic outcome. This dual corroboration of the SHAP and nomogram analyses not only enhances the credibility of our findings but also provides clinicians with an intuitive, explainable, and auxiliary diagnostic tool for MD.

This study has three main limitations. First, the data was sampled from students at a single university, which limits its regional scope. Second, the model was built on a relatively small dataset of 1,379 healthy and patient individuals. Third, comorbidity information of the participants was not collected or recorded in this study, and relevant comorbidity factors were not included in the prediction model, which may exert potential confounding effects on the predictive results of the model. Based on these limitations, future research should focus on the following improvements. (1) Enhance generalizability: Future studies should include diverse populations from different regions and age groups to improve the universal applicability of the findings. (2) Improve robustness: The sample size should be expanded for large-scale, multi-center studies to improve the model’s robustness and generalization capability. (3) Future relevant studies should consider incorporating comorbidity as a covariate into model analysis to further improve the accuracy and reliability of the prediction model.

## 5 Conclusion

This study successfully developed a MD prediction model based on the XGBoost algorithm and CBC variables. This model cannot only assist clinicians in the early identification of high-risk individuals for MD, but also lays the groundwork for research on MD biomarkers, providing a new direction for future exploration in the field of mental health.

## Data Availability

The raw data supporting the conclusions of this article will be made available by the authors, without undue reservation.
